# Clinical, Radiological and Histopathological Analysis of Metastatic Tumors of the Oral and Maxillofacial Region: A Retrospective Case Series

**DOI:** 10.7759/cureus.105805

**Published:** 2026-03-24

**Authors:** Anjitha V A, Ramesh S, Tinky C Bose, Nihala P A, Fathima Shirin C R

**Affiliations:** 1 Department of Oral Medicine and Radiology, Government Dental College, Thiruvananthapuram, Thiruvananthapuram, IND; 2 Department of Oral Medicine and Radiology, Indira Gandhi Institute of Dental Sciences, Kothamangalam, IND

**Keywords:** adenocarcinoma, breast cancer, lung cancer, mandible, maxilla, oral metastatic tumors, paresthesia, thyroid cancer, unilocular radiolucencies

## Abstract

Aim

Oral metastatic tumors are rare and may present as vague pain or painless swellings and are misdiagnosed as odontogenic pathologic entities such as odontogenic cysts, tumors, pulpal or periapical diseases, etc. Since oral metastatic tumors are uncommon, the diagnosis of oral metastatic tumors is challenging for clinicians and pathologists. In this study, we aimed to analyze the clinical, histopathological, and radiological characteristics of oral metastatic tumors to assist clinicians and pathologists in achieving early diagnosis and proper management.

Materials and methods

The clinical, histopathological, and radiological characteristics of patients diagnosed with metastatic tumors of the oral cavity over the past 10 years at the Department of Oral Medicine and Radiology in Government Dental College, Thiruvananthapuram were retrospectively collected and analyzed. We analyzed complete medical and dental records of 1434 patients to assess the prevalence of oral metastatic tumors. A detailed analysis of clinical and radiological features of metastatic tumors was done by Oral Medicine and Radiology specialists. The primary tumor site and histopathological features were also evaluated.

Results

Among 1434 cases, 10 (0.69%) cases of oral metastatic tumors with equal gender distribution (five male patients & five female patients) and a mean age of 54 years were obtained. In our retrospective study, the mandible was the most frequent site of metastases, and the most frequent complaint was painless swelling. Our findings demonstrated a consistent relationship between specific primary sites and gender, notably lung cancer in male patients and breast and thyroid cancer in female patients. Adenocarcinoma was the most frequent histological diagnosis, accounting for 50.0% (five out of ten cases) of the cases, and unilocular radiolucency was the most prevalent radiological presentation.

Conclusion

Our study showed that oral metastatic tumors frequently masquerade as benign conditions, most commonly presenting as painless swelling and unilocular radiolucencies. The high predilection for the posterior mandible (70%) and the significant association between specific primary sites and gender, notably lung cancer in male patients and breast/thyroid cancer in female patients, highlight the need for a high index of suspicion. Ultimately, as metastatic tumors of the oral cavity often mimic common dental pathologies, clinicians must prioritize thorough historical review and histological evaluation, particularly in older patients, to ensure early diagnosis and appropriate management of secondary malignancies.

## Introduction

Carcinoma is characterized by loss of normal cellular balance, leading to uncontrolled growth and an unnatural ability for cells to survive. In the clinical management of carcinoma, the spread of the disease to distant sites, known as metastasis, is the most significant factor affecting patient health and survival rates. This process is not a single event but a complex series of biological steps. Mandibular metastatic tumors are more prevalent than maxillary metastatic tumors. These tumors can present in two distinct ways: by infiltrating the oral soft tissues or by developing centrally within the jaw bones [1].

The oral metastasis is often linked to poor prognosis, so early diagnosis is mandatory. The current literature largely comprises isolated case reports, which may introduce bias by over-representing atypical manifestations. Sometimes, oral metastatic tumors may present as vague pain and be misdiagnosed as pathologic entities of dental origin, such as pulpal or periapical diseases, odontogenic cysts, tumors, etc. Recently, a case of an oral metastatic tumor from breast carcinoma mimicking a periodontal abscess in the mandible has been reported. The rarity of oral metastatic tumors makes their diagnosis difficult for clinicians and pathologists [2]. This study comprises 10 cases of oral metastatic tumors from thyroid, breast, lung, colon, and prostate carcinoma. Evaluation of the clinical, radiological, and histopathological profiles of patients with oral metastatic tumors to enhance our knowledge about their presentation were done. By standardizing these findings, we aim to assist practitioners in maintaining a high index of suspicion for secondary malignancies in the oral and maxillofacial regions.

## Materials and methods

In this retrospective case series, 1434 patients diagnosed with oral malignant tumors over the past 10 years at the Department of Oral Medicine and Radiology in Government Dental College, Thiruvananthapuram were enrolled. Only those cases with complete clinical, histopathological, and radiological documentation were included. Following Institutional Ethics Committee approval, relevant clinical, radiological, and histopathological data from existing medical records were extracted and analyzed to assess the prevalence of oral metastatic tumors. A detailed analysis of clinical and radiological features of metastatic tumors was done by Oral Medicine and Radiology specialists. The primary tumor site and histopathological features were also evaluated.

**Figure 1:**
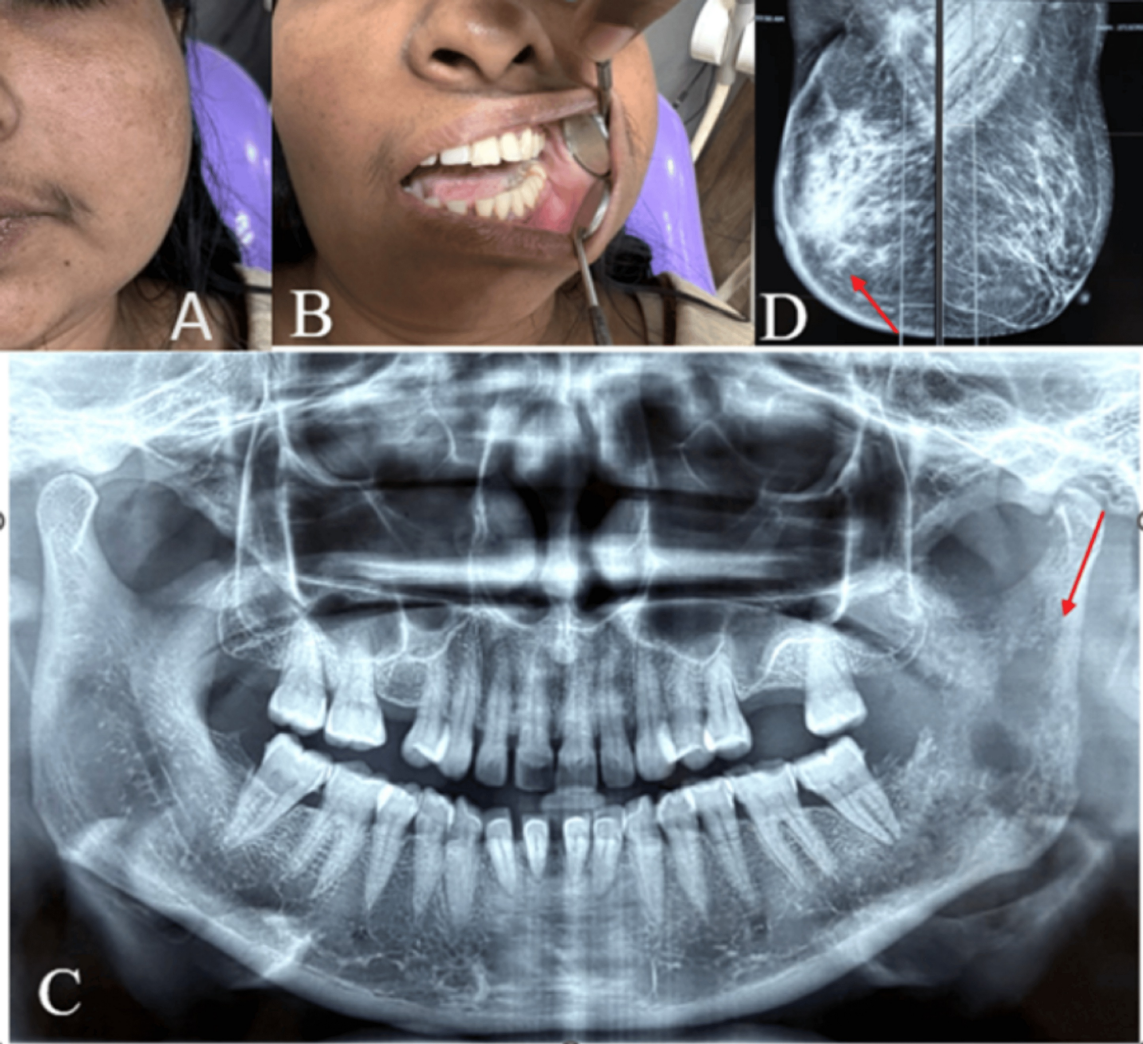
Mandibular metastasis of breast carcinoma. A: Extraoral picture shows a diffuse swelling on the left lower jaw. B: Restricted mouth opening. C: Panoramic radiograph shows multiple ill-defined lytic areas. D: Mammogram shows an irregular high-density lesion in the retroareolar region of the right breast

**Figure 2:**
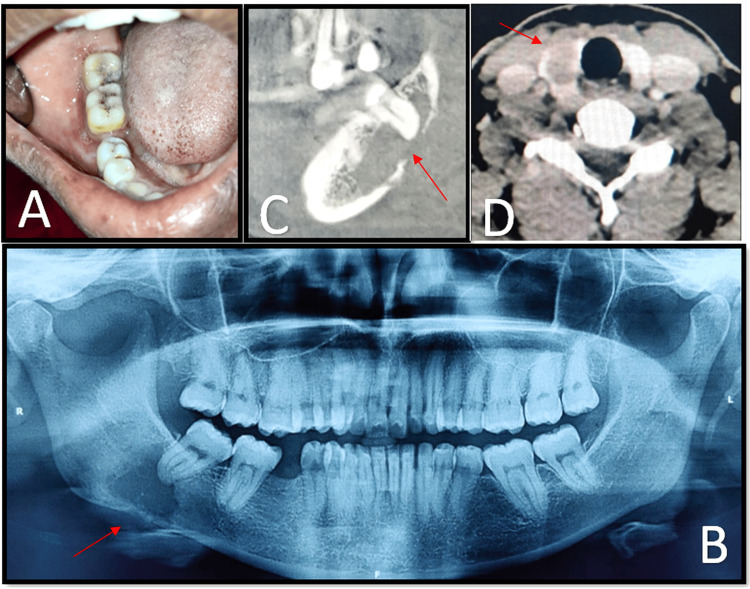
Mandibular metastasis of papillary carcinoma of thyroid. A: 2 x 1 cm swelling noted on the mandibular vestibule region in relation to 48. B: Panoramic radiograph showing a unilocular osteolytic lesion with irregular borders (red arrow) measuring about 2 x 3 cm in the posterior mandible in relation to the periapical region of 48. C: Sagittal section of CBCT shows an osteolytic radiolucent lesion with irregular borders on the right body of the mandible (red arrow) in the periapical region of 48. D: CECT head & neck revealed a relatively well-defined hypoattenuating mass of about 12 x 18 mm (red arrow) in the right lobe of the thyroid gland.

Sociodemographic details of patients, clinical features, radiological features, primary tumor location, and histological features were analyzed in this study using IBM SPSS Statistics for Windows, Version 21 (Released 2012; IBM Corp., Armonk, New York, United States). Descriptive statistics was done for all outcomes and study variables. Outcome variables were expressed as proportions or percentages with 95% CI. Numerical variables were expressed as mean and standard deviation. Association between outcome variables and study variables was tested using the chi-square test. p-values less than 0.05 were taken as statistically significant. All study procedures were in accordance with the Helsinki Declaration [3] and GCP guidelines [4]. Figures 1, 2 represent two different cases of oral metastatic tumors.

## Results

Prevalence, age, and gender distribution

A total of 1434 cases of oral malignant tumors reported over the last 10 years at our hospital were included: 10 cases of oral metastatic tumors, 20 cases of sarcomas, six cases of melanomas, and 1398 cases of carcinoma. The proportion of oral metastatic tumors accounted for 0.69 % of all oral malignant tumors. The study exhibited an equal gender distribution (five male patients and five female patients) with a mean age of 54 years (range: 38-71 years).

Clinical features of metastatic carcinoma

**Figure 3:**
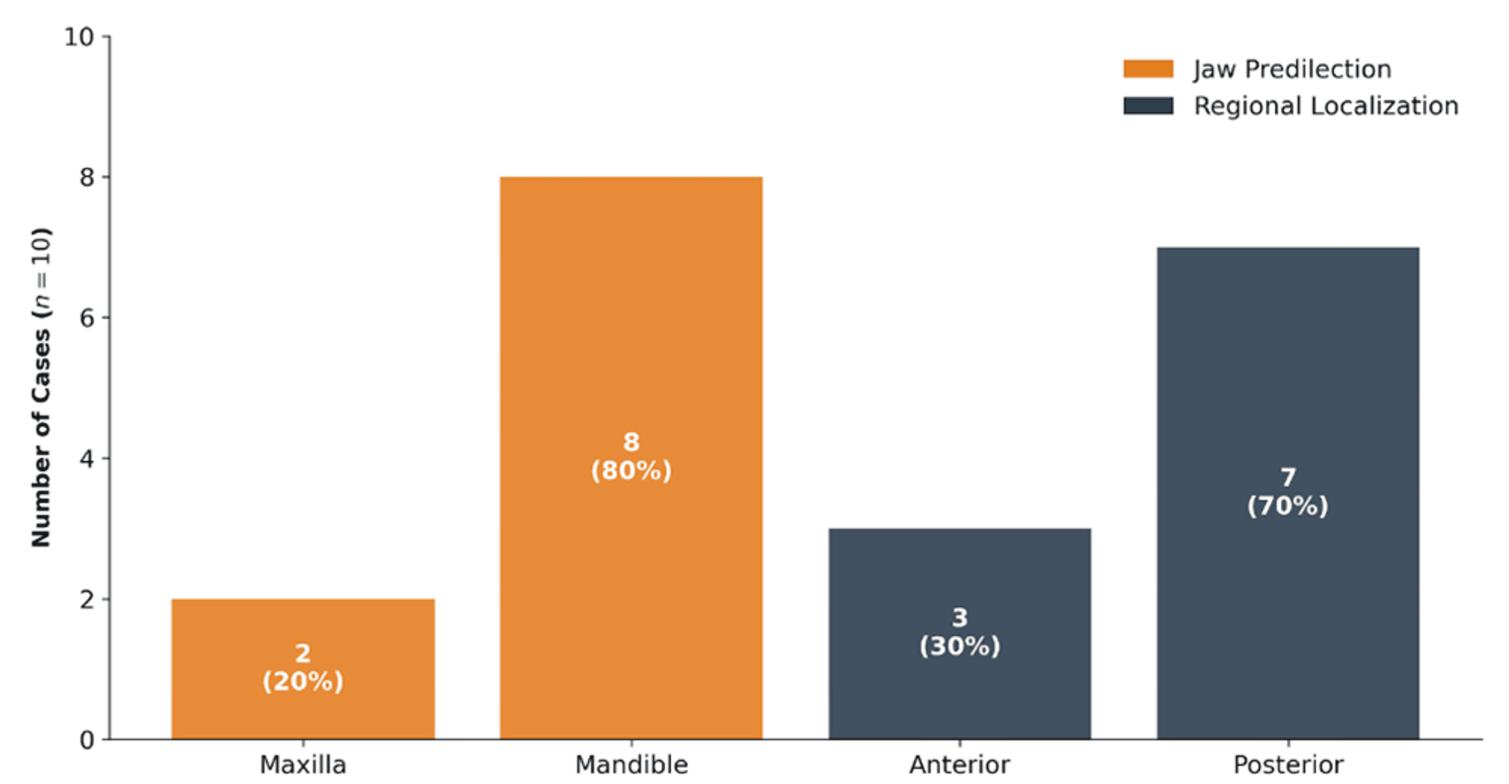
Jaw predilection and regional localization

The mandible was the most frequent site for metastases, accounting for 80% (eight cases) of cases, whereas the maxilla accounted for only 20% (two cases). There was a strong preference for the posterior regions of the oral cavity, which represented 70% (seven cases) of the locations, compared to 30% (three cases) in the anterior regions (Figure 3).

The clinical presentations varied, but painless swelling was the most common symptom, observed in 50% (five cases) of the study population; 30% (three cases) of patients experienced a combination of pain and swelling. Sensory disturbances were a notable feature, with 10% (single case) of patients reporting paresthesia and another 10% (single case) reporting a complex of pain, numbness, and swelling.

Radiological features

**Figure 4:**
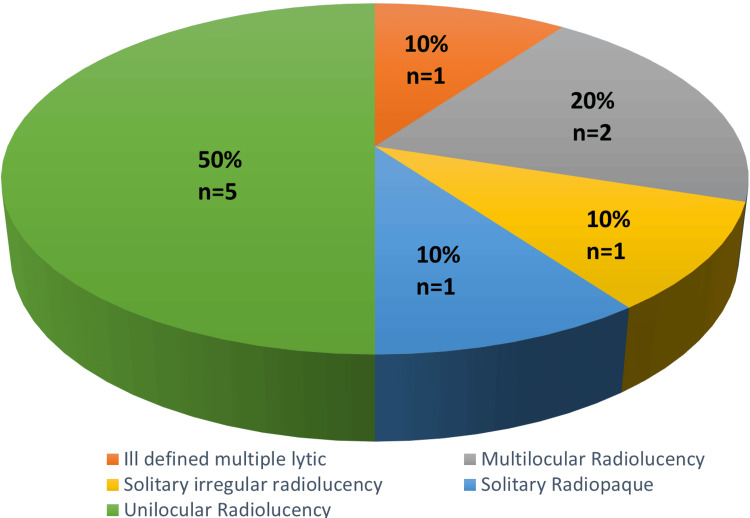
Radiological features

Unilocular radiolucency was the most prevalent radiological presentation, occurring in 50% of cases. Multilocular radiolucency a less common but significant presentation, observed in 20% of the study population; 10% (single case) of cases exhibited aggressive, ill-defined multiple lytic lesions and another 10% (single case) appeared as solitary irregular radiolucencies, while a single notable case (10%) presented as a solitary radiopaque lesion (Figure 4).

Primary site distribution

**Figure 5:**
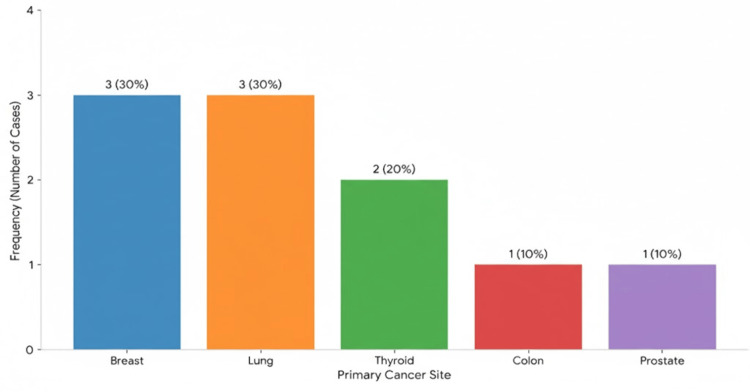
Distribution of primary sites

Breast and lung cancers were the most frequent primary sources, each accounting for 30% of the oral metastases (three cases each). Thyroid was the next most common origin, contributing to 20% of the cases (two cases). Single instances (10% each) were identified originating from the colon and the prostate (Figure 5).

Histological features

**Figure 6:**
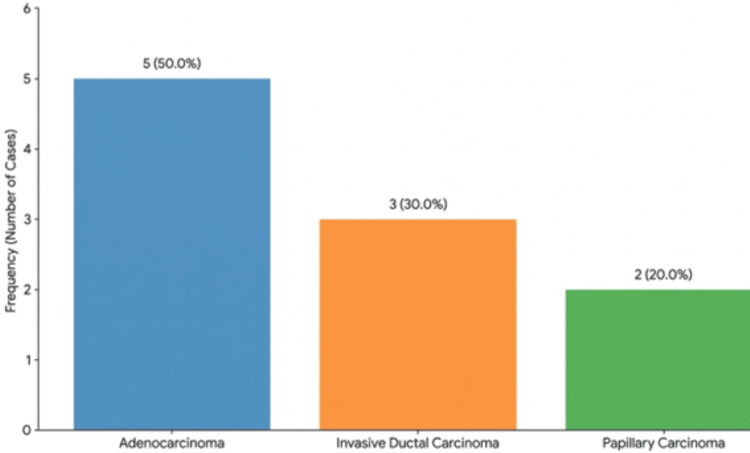
Distribution of histological features

Adenocarcinoma was the most frequent histological diagnosis, accounting for 50.0% of the cases (five cases). Invasive ductal carcinoma accounted for 30.0% of the cases (three cases), and papillary carcinoma was seen in 20.0% of the study population (two cases) (Figure 6).

Association of gender with clinical, radiological, primary tumor, and histological features

There was a clear gender divide based on the origin of the primary tumor. Breast and thyroid tumors were found in female patients (100%), and lung, prostate, and colon tumors were found exclusively in male patients (100%).

The microscopic nature of the tumors differs significantly by gender. Adenocarcinoma was observed only in male patients (100%) in our study. Invasive ductal carcinoma and papillary carcinoma were observed exclusively in female patients (100%).

Gender did not appear to have a statistically significant impact on the location of oral metastatic tumors, clinical signs and symptoms, and radiological features.

Comparison of age with clinical, radiological, primary tumor, and histological features

A statistically significant difference was seen in the mean age of patients based on the primary tumor sites (p = 0.032). Patients with prostate cancer (mean age 70.00 years) and lung carcinoma (mean age 65.67 years) were generally older. Patients with carcinoma of the colon presented at a notably younger mean age of 38.00 years. Patients with carcinoma of the breast had a mean age of 48.00 years.

No significant correlation was found between age and jaw distribution (p=0.557), location (p =0.909), clinical signs and symptoms (0.605), radiological features (p=0.684), and histological features (p=0.214).

Association of clinical features with radiological features

Painless swelling was the most frequent clinical presentation (five cases), primarily associated with unilocular radiolucency (60%) and multilocular radiolucency (40%). Patients presenting with both pain and swelling (three cases) most commonly exhibited unilocular radiolucency (66.7%), followed by ill-defined multiple lytic lesions (33.3%). The single case of paresthesia (one case) was exclusively associated with a solitary irregular radiolucency (100%). A solitary radiopacity presented with a combination of pain, numbness, and swelling.

Association of clinical features with histological features

Painless swelling was the most frequent clinical presentation (five cases), with adenocarcinoma being the most common histological finding in this group (60%). Patients presenting with both pain and swelling (three cases) showed an even distribution across all three histological types: adenocarcinoma (33.3%), invasive ductal carcinoma (33.3%), and papillary carcinoma (33.3%). The single case presenting with this combination of pain, numbness, and swelling was identified as adenocarcinoma (100%). The single case presenting with paresthesia was identified as invasive ductal carcinoma (100%).

Association of clinical features with the primary tumor site

Painless swelling (five cases) was observed across a variety of primary sites: lung (40.0%), breast (20.0%), colon (20.0%), and thyroid (20.0%). Patients presenting with the combination of pain and swelling (three cases) were evenly distributed (33.3% each) among those with breast, lung, and thyroid primary tumors. The single case presented with pain, numbness, and swelling originated from a prostate primary tumor. The single instance of paresthesia was associated exclusively with a breast primary tumor.

Association of radiological features with the primary tumor site

Among all primary tumor types other than prostate, unilocular radiolucency was the most frequent radiological feature (five cases). It was most common in lung carcinoma cases (40%). Multilocular radiolucencies were seen only in lung (50%) and thyroid (50%) carcinoma cases. Solitary radiopacity was unique to the prostate carcinoma (single case). Both ill-defined multiple lytic and solitary irregular radiolucencies were observed exclusively in breast carcinoma cases within this study sample.

Association of histology with the primary tumor site

Adenocarcinoma was the most diverse histological type, appearing in cases of the lung (60%), colon (20%), and prostate carcinoma (20%). Invasive ductal carcinoma was exclusively associated with breast carcinoma, representing 100% of the cases within that category. Papillary carcinoma was found exclusively in the thyroid, representing 100% of the cases within that category.

Association of the jaw with the primary tumor site

Mandibular metastasis was most commonly seen in patients with breast carcinoma, followed by lung and thyroid carcinoma. Colon and lung primary tumors presented with metastatic tumors in the maxilla, accounting for 50.0% of maxillary cases each.

Association of the location with the primary tumor site

The posterior region was the most common site, with lung cancer being the most prevalent primary source (42.9%), followed by breast and thyroid carcinoma (28.6% each). Breast, colon, and prostate carcinoma produced anterior metastatic tumors, with each primary site representing 33.3% of the anterior cases (three cases).

## Discussion

Based on the study results, 10 cases of oral metastatic carcinoma were analyzed, and they demonstrated a balanced gender distribution and a mean patient age of 54 years. The mandible was the primary site of involvement, accounting for 80% of cases, with a significant 70% predilection for the posterior oral cavity regions. Clinically, presentations were led by painless swelling (50%), while radiological findings most frequently revealed unilocular radiolucency (50%). Breast and lung tumors were the most common primary sources (30% each), followed by thyroid carcinoma (20%), with significant gender-specific correlations: breast and thyroid tumors were found exclusively in female patients, whereas lung, prostate, and colon tumors were found only in male patients. Histologically, adenocarcinoma was the most prevalent diagnosis at 50%, occurring solely in male patients within this dataset.

In our study, we included 10 cases of oral metastatic tumors, and the proportion of metastatic tumors accounted for 0.69% of all oral malignant tumors, whereas the study by Lee et al. included 21 cases of oral metastatic tumors and the proportion of metastatic tumors accounted for 1.03% of all oral malignant tumors. The findings of this study, which indicated a mean age of 54 years, align closely with the mean age of diagnosis of 56.9 years from the study by Leeet al.(2017) [2]. While this study demonstrated a balanced gender distribution for oral metastatic tumors, the study by Lee et al. showed male predilection, and the study by Hirshberg et al. showed a slight female predilection [5]. Similar to the Irani (2020) study, our study also showed a strong preference for the mandible over the maxilla [6-8]. The study by Lee et al. (2017) and our study found that 70% of metastases occurred in the posterior segments. This preference is largely attributed to the robust vascularity of the posterior mandible, which provides an ideal pathway for tumor cell seeding and colonization [2,9].

The clinical symptoms of oral metastases in this study were diverse, with painless swelling emerging as the most frequent symptom (50%), which is consistent with the findings of Irani (2020), who noted that a significant portion of metastatic tumors present as asymptomatic expansions, often leading to delayed diagnosis or misidentification as benign tumors [6,10]. Although paresthesia was observed in only one case in this cohort, its presence remains a classic red flag for malignancy in the mandible, as it indicates perineural invasion or pressure on the inferior alveolar nerve. Similar to the study by Lee et al. (2017), unilocular radiolucency (50%) was our most prevalent radiographic finding, which highlights the diagnostic challenge oral metastatic tumors pose [2,11-15].

Consistent with the study by Jham et al., our study identified lung carcinoma as the primary source of metastasis in men. For women, while Jham et al. reported breast and lung as the leading sites, our findings showed a predominance of breast and thyroid tumors. In both studies, adenocarcinoma was the most frequent histological diagnosis (50%), reflecting the primary origins found in this study (breast and lung) [1,16,17].

The study is limited by a small cohort of only 10 cases, which may not be the true representation of the diverse clinical spectrum of oral metastatic carcinomas. Moreover, the data was collected only from the Department of Oral Medicine & Radiology at Government Dental College, Thiruvananthapuram, potentially limiting the findings to a specific geographic and demographic population. Since it was a retrospective chart review, there may be a possibility of missing data or information bias. Also by focusing strictly on cases with full clinical and imaging data, we may have limited our ability to capture the soft-tissue metastatic presentations.

## Conclusions

Our study results suggested that oral metastatic tumors frequently masquerade as benign conditions, most commonly presenting as painless swelling and unilocular radiolucencies. The high predilection for the posterior mandible (70%) and the significant association between specific primary sites and gender, notably lung carcinoma in male patients and breast/thyroid carcinoma in female patients highlight the need for a high index of suspicion. Ultimately, because as metastatic tumors of oral cavity often mimic common dental pathologies, clinicians must prioritize thorough historical review and histological evaluation, particularly in older patients, to ensure early diagnosis and appropriate management of secondary malignancies.

Future directions should prioritize multi-institutional data collection to expand sample sizes and provide a more diverse global overview of oral metastases. Researchers must also consider data regarding patient habits, such as tobacco and alcohol use, to identify potential lifestyle correlations. Finally, incorporating details on soft tissue involvement will allow for a more precise characterization of the clinical profile of these secondary malignancies.
